# Motivating Military Trainee Healthy Eating: Insight from Two Sites

**DOI:** 10.3390/foods9081053

**Published:** 2020-08-04

**Authors:** Anna Kitunen, Julia Carins, Sharyn Rundle-Thiele

**Affiliations:** 1 Social Marketing @ Griffith, Department of Marketing, Griffith University, 170 Kessels Road, Nathan, QLD 4111, Australia; j.carins@griffith.edu.au (J.C.); s.rundle-thiele@griffith.edu.au (S.R.-T.); 2 Food and Nutrition, Land Division, Defence Science and Technology, Scottsdale, TAS 7260, Australia

**Keywords:** eating behaviour, eating motivations, young adults, military trainees, behaviour change, segmentation

## Abstract

This paper investigates eating behaviours and motivations of early career military trainees from two pathways (officer cadets and army recruits) to understand whether, and to what extent, healthful eating behaviours were present, and what motivates eating in general and healthful habits specifically. The study also sought to understand whether groups need strategies that are pathway specific or are transferrable across different trainee populations. Participants were recruited via email to complete an online survey and through in-person invitations to ensure a diverse cross section of trainees (n = 195) and recruits (n = 297). Two-step cluster analysis revealed three distinct segments with education, opportunity and motivation being the most important variables within a wider multivariate segment formation and stepwise linear regression was performed to identify the most optimal model with the least number of predictors for each segment. The ideal model for the uninterested segment contained nine predictors, seven predictors for the Breakfast skippers segment and eight predictors for the Weight conscious segment. This study found that there is room for improvement in the eating habits of military trainees across military training pathways. Eating motivations, and their associations with healthful eating habits, indicate a need for strategies that are broader than instilling or reinforcing health motivations. Strategies that can be implemented to support healthful eating for military trainees include provision of food preparation and cooking skills training, coupled with provision of social support and environments that facilitate healthful eating. Furthermore, three distinct segments were discovered within the trainee population, indicating that strategies for positively changing trainees’ eating behaviours may not need to be pathway specific, rather it may be possible to introduce the same group of segmented strategies across both pathways. Future research directions and limitations are outlined.

## 1. Introduction

Consumption of a nutritious diet is essential for health and performance [1,2]. For military personnel to perform their roles well, it is important they occupy the positive end of the health and performance continuum requiring them to establish and maintain healthful nutritional practices. When seen as a continuum, a nutritious diet has a key role in preventing a breakdown in health (avoiding illness and disease), allowing normal functioning (physical, mental and emotional wellbeing), facilitating restoration of health (enhancing healing from injury), and enabling peak performance (optimisation of functioning).

To pursue optimised performance and to accomplish military readiness, there is a growing recognition of the need for a comprehensive view of health and performance [3,4]. These wider views recognise the role of the body, the mind, and the team. In terms of the physical element, inadequate nutrition is associated with health and physical performance [5], whereas healthful diet is linked with higher fitness levels [2]. The prevalence of illness and injury increases due to overnutrition (overweight and obesity), which reduces productivity and increases healthcare costs [6]. The effect of nutrition on the cognitive element is also clear, with suitable nutrition being essential for cognitive performance [7], mental health [8] and resilience [2]. Foods and the different locations in which foods are consumed also have a social and cultural element, providing opportunities to share, bond, and partake in traditions and rituals [9] specific to a group or culture. Given the importance of nutrition within a comprehensive model of performance and health, and evidence of suboptimal eating [10,11,12], strategies are required to embed and strengthen the drive to instil healthful eating behaviours in military personnel.

Young adulthood (18–25 years) is a crucial life-stage during which long-term health behaviour habits are formed [13]. During this foundational life-stage many personal, social and environmental changes happen such as entering tertiary education, moving away from home from parentally-assisted to independent decision-making, moving in with partners or peers, and entering the workforce [14]. These changes have been linked to poor lifestyle behaviours such as consumption of an unhealthy diet [15], sedentary lifestyles [16,17] and weight gain [18]. Therefore, it is essential to form healthful eating habits while young [19] and the foundational life-stage presents a window of opportunity for improving young adults’ eating habits [20]. The majority of Australian Defence Force (ADF) trainees (~85%) are within the age range of 18–25 years [21] and their training can serve as a catalyst for personal change [22], given the changes in living arrangements and work responsibilities. As trainees join the military, they start a new career and new lifestyle; for many, military training creates a new persona. Recruits go through a “reset” or “remoulding” phase where a military identity is formed and their civilian status is dissolved [22]. Transitions from one life-stage to another involve a high level of change and disruption, which destabilises previously formed behavioural patterns, creating room for new habits to form [23]. It is crucial to understand eating behaviour and behavioural influences in this group, given it can serve as a baseline that can be utilised to reset poor habits and to support and reinforce existing healthful eating habits, which are considered essential for optimal performance. Considering this baseline, strategies that encourage the formation of healthful eating behaviours to support health and performance can be implemented. Formation of new habits early on in a transitioning life-stage carries the likely prospect that these habits could persist for life, producing benefits for individuals throughout their military careers, for the military organisation and for society more broadly.

This study aimed to understand eating behaviours and factors influencing eating behaviours. This study examined eating behaviours and motivations for eating within ADF trainees from two pathways who are at the transitioning life-stage of commencing a military career. Examining two pathways provides understanding of the preferences and needs of each group and enables consideration of whether strategies are required to be pathway specific or transferable across the different trainee populations.

## 2. Conceptual Foundation

Motivation is crucial for deliberate behaviour and provides the drive to act in a specific way [24,25]. Motivation can be considered as a starting point with a variety of other factors influencing whether motivation is translated into action. Ability to perform a behaviour is required [26] and the social influences of others might encourage or restrain behaviour [27]. Furthermore, physical environments influence behaviour through opportunity—by providing either facilitative or prohibitive elements or conditions that trigger spontaneous behaviour [28,29]. This study utilised the Motivation, Opportunity and Ability (MOA) framework [30] and aimed to capture eating behaviours known to contribute to health, and assign those to the eating motivations, ability and opportunity of ADF trainees.

The MOA framework has been utilised to categorise individuals into distinct groups based on how prone, resistant or unable the individual is to change depending on their motivation, opportunity and ability to perform the specific behaviour [30]. Motivation is comprised of the internal drivers to perform a behaviour. Motivation contains interest, readiness, willingness and desire to engage in information processing [31] or a particular behaviour [32]. It has been suggested that perceived risk and alignment with existing attitudes, as well as needs and goals impact motivation [33]. Ability refers to the extent that people have the needed skills or capabilities to engage in particular behaviour to reach the outcome [32,33]. Self-efficacy [26] is a determinant of ability [30] and includes an individual’s beliefs regarding their skills to perform and organise a series of actions [26]. Environmental influences are likely to impact self-efficacy, such as cultural norms, and self-efficacy has been associated with motivation to perform a behaviour [34]. Opportunity is the extent to which external influences and factors prevent or mitigate engaging in a particular behaviour [32]. Accordingly, lack of opportunity includes situations where an individual can be motivated to perform a behaviour but is prevented from doing so due to environmental factors [30] (e.g., low or lack of supply of healthful alternatives).

The MOA framework has been previously used within the weight management context (nutrition and physical activity) where the influence of interrelated motivation, opportunity and ability on the behavioural outcomes among Australian adults were examined [35]. The results showed that 63% of the participants who reported changes to their mindset or forming new habits, reported weight loss after participation in the program. Furthermore, the findings indicated a significant relationship between changes adopted and new habits formed from participation in the weight management program and participant’s wellbeing and health outcomes including weight loss following participation in the program [35]. This indicates that the MOA framework is suitable for improving understanding of the market for changing eating behaviour.

This study also utilised segmentation, which is an analytical approach commonly used in both marketing and social marketing, to determine whether smaller groups with similar characteristics exist within a larger target audience [36], as well as offering a means to understand the different wants, needs and characteristics of those groups. Segmentation finds main groups and enables prioritisation of resources based on factors such as size and receptivity of the segments [37]. Research shows that segments respond to social marketing interventions differently [38] suggesting that better outcomes may be reached when different strategies designed for the different segments are included in the program, hence offering solutions that benefit each segment.

Programs could be delivered without the use of segmentation, utilising a one size fits all approach that assumes that all trainees possess similar wants and needs [39]. However, a recent study found that a one size fits all approach failed to positively change young adults’ eating behaviours [40]. Program effectiveness, therefore, may be limited when a one size fits all approach is applied given that large numbers of the target audience may be left uninterested, unchallenged, or dissatisfied [41]. Discovering segments within a larger group of trainees allows for the design of strategies tailored to those segments, which avoids the ineffectiveness of a one size fits all approach, and also the potentially costly nature of one-to-one implementation approaches. Moreover, segments that are not based on the trainee pathway (or location) might be found, indicating that it might be possible to introduce the same segment strategies at different locations.

## 3. Materials and Methods

### 3.1. Target Population

A survey was administered to participants from two trainee pathways—officer cadets at the Australian Defence Force Academy (ADFA) in Canberra and army recruits at the Army Recruit Training Centre (ARTC) in regional New South Wales. The survey was completed online at ADFA, and on paper at ARTC (due to limited computer access). Participation in the survey was voluntary and completely anonymous, and the survey took approximately 15 min to complete. This study was a part of a collaborative research project between the university and a Government research organisation. Ethical approval for the study was obtained through the Human Research Ethics Committees of both organisations (approval code 2017/504).

### 3.2. Survey

The survey measured eating habits known to contribute positively and negatively to health. The first section of the survey assessed eating behaviour related specifically to breakfast intake, number of meals consumed on a daily basis, the level of vegetable and fruit consumption on a daily basis, daily consumption of both alcohol and desserts, and daily water intake. Each eating habit was scored on a scale from “Never” to “Always” (0 to 3) and eating habits were combined to create a score to measure the healthfulness of eating habits (range 0 to 24) [42]. The second section of the survey captured motivations for eating (healthfully or not) on a seven-point Likert scale with options ranging from “Never” to “Always” in the format “I eat what I eat… because I am hungry” (for further examples, see Appendix A for a full list of motivational items). Items included internal motivations for eating such as hunger, need for energy, need for satiety, health, weight control and convenience. Items for external influences included cooking ability, time, opportunity, sociability, social image, conventional eating and social norms [43]. In the final section of the survey, participants were asked a number of demographic questions capturing age, gender, education level, height and weight.

### 3.3. Data Analysis

Descriptive statistics were calculated, including frequencies and percentages for demographic and behavioural variables. The reliability and validity of the eating motivations constructs were examined. Two-step cluster analysis was performed [44] to detect homogenous segments within the heterogeneous study population. Previously, this method has been used in identifying segments in healthful eating behaviours [45], adolescent populations [38] and in physical activity behaviours [46,47]. The analysis is an appropriate method for this study given that the numbers and members of the segments are unknown [48]. Furthermore, it allows simultaneous analysis of both categorical and continuous data providing an ability to simultaneously address a wide and diverse range of measures and the capability to handle large sample sizes [44,48]. The analysis was executed with five variables with zero to low correlations and the measure-to-respondent ratio was within the recommended levels [49]. Segment solution was validated in a split sample to warrant the consistency of the segment formation in a half-sized sample. One-way ANOVA tests and Chi-square tests were executed on the categorical and continuous variables to examine segment differences. A threshold of *p* < 0.05 was used to determine statistically significant differences. Stepwise linear regression with backward elimination was conducted to examine relationships between the healthfulness of eating habits (as the dependent variable) and motivations and/or influences (as the independent variables). The most parsimonious model with the most explanatory power was identified by optimising R^2^ (explanatory power) with adjusted R^2^ (explanatory power adjusted for the number of predictions). All statistical analyses were conducted with IBM SPSS Statistics version 25.

## 4. Results

### 4.1. Sample

A total of 632 military trainees responded to the survey and of those 492 (195 responses from officer cadets and 297 from recruits) were used in the analysis after data cleaning. In total, 140 responses were removed due to incomplete data or failure to correctly answer the attention check question. Most respondents were aged 18–25, and for both trainee pathways more than half of the respondents were male. The officer cadet group was younger (cadet mean = 19.9, recruit mean = 23.1, t = 10.130, *p* < 0.001) and contained more females than the recruit group (cadet 32.3% female, recruit 20.5% female, x^2^ = 9.153, *p* < 0.002). The officer cadet group had a higher proportion of high school educated respondents when compared to the recruit group (cadet 86.8%, recruit 77.4%, x^2^ = 6.536, *p* < 0.011), and similarly a higher proportion of respondents in a healthy weight range (cadet 72.8% healthy Body Mass Index (BMI), recruit 59.9% healthy BMI, x^2^ = 8.599, *p* < 0.003). See Table 1 below.

The healthfulness of eating habits score was lower for officer cadets than recruits (cadet mean = 6.7, recruit mean = 16.9, t = 33.958, *p* < 0.001). The lower healthfulness of eating habits score for cadets was due to lower breakfast consumption, water and vegetable consumption, higher alcohol and dessert consumption, and less regular meal consumption. Both dairy and fruit consumption levels were similar between cadets and recruits. Within the officer cadet population, 47% reported never consuming two portions of vegetables per day. This places the cadets at risk of not receiving the vital nutrients for performance and health through their daily meal intake. Furthermore, more than half of the cadets (65%) reported never eating breakfast, 60% reported never eating three meals per day, and 61% reported never consuming 1–1.5 L of water on a daily basis. There is a possibility that some cadets may have adopted a meal consumption pattern that includes more than three meals per day, however, at face value, these habits are not optimal. More than half of the cadets also reported consuming cakes or desserts, or alcohol at meals either always or often. These eating habits can further increase health risk when performed on a regular basis. In the army recruit population, the number of recruits that reported never performing any of the healthful eating behaviours was low. See Table 2.

### 4.2. Eating Motivations

Two of the eating motivations constructs (Need and Hunger, and Social Norms) [43] did not demonstrate high internal consistency and failed to exceed the recommended Cronbach’s alpha levels, indicating individual survey items might be measuring separate constructs. Further testing showed that all three items under the Need and Hunger construct should be considered as individual constructs (Hunger, Need for Energy and Pleasantly Filling—termed satiety) and these could not be combined into one construct. Social Norms formed a construct with two items but the third item (Conventional Eating) was considered separately. All items and constructs are reported in Appendix A. All other constructs demonstrated high internal consistency and approached or exceeded the recommended Cronbach’s alpha levels (α = 0.70). Participants reported motivations for eating the foods they do (healthful or not) and these motivations are considered to support eating choices for both healthful and unhealthy foods. For both cadets and recruits, the strongest motivations for eating were need for energy, hunger, convenience, health and satiety. Social image and social norms were the weakest motivations for eating for both groups. See Table 3 below for detail for each group.

Some group level differences were observed between cadets and recruits. Recruits reported higher levels of motivation for eating for convenience, satiety, opportunity, ability, social norms and weight control, while cadets indicated stronger motivations for eating were due to sociability and lack of time. Even though all of the differences between the groups were significant, in most instances the magnitude of the differences was less than one point on the scale. The only exception was lack of time with more than one-point difference on the scale.

### 4.3. Behavioural Segments

Two-step cluster analysis produced a sample (n = 492) with a silhouette measure of cohesion and separation of 0.2, which aligns with other segmentation studies [50]. For the differences between and within segments to be valid, a silhouette measure of more than 0.0 is required [44]. A three-segment solution was generated with five segmentation variables followed by the evaluation of variable predictor importance scores (from 0 = least important to 1 = most important). The most important variable defining the segments was education (1.00), followed by opportunity (0.51), motivation (0.50), ability (0.43) and the healthfulness of eating habits score (0.13) as the least important variable. The split sample test generated a consistent segment solution with the original sample with a silhouette measure of cohesion and separation of 0.2 and a three-segment solution (1.00 = education, 0.69 = motivation, 0.46 = opportunity, 0.40 = ability and 0.32 = the healthfulness of eating habits score).

The segments were named Uninterested, Weight conscious and Breakfast skippers, based on their lower, middle and higher levels of motivation, respectively. The levels of motivation in each segment corresponded with lower, middle and higher levels of opportunity, ability and healthfulness of eating habits score. The first segment (Uninterested, 38% of the entire sample) was high school educated (100%), contained more cadets (59%), was younger (M = 20.4 years) and was mostly male (77%). The second segment (Breakfast skippers, 19% of the entire sample) contained degree educated members (36.6%), consisted of mostly recruits (72%), was older (M = 25.6 years), and whilst mostly male (66%), the segment contained more females than the other two segments. The third segment (Weight conscious, 43% of the entire sample) was high school educated (100%), consisted of mostly recruits (73%), was between the other segments in age (M = 21.6 years) and was also mostly male (76%). No significant differences were observed between the segments in terms of the proportion of respondents in the BMI categories (underweight, normal weight and overweight). See Table 4 below for detail for each segment.

After the three segments were formed, the motivations for eating within each segment were compared. The motivations for eating between the three segments did not differ for the scales of hunger and convenience, social norms and sociability. However, the Uninterested segment was significantly different from the other two segments reporting higher levels for lack of time and lower levels for satiety. This indicates that the respondents in the Uninterested segment are less motivated to choose foods because the foods are filling, and more motivated to choose foods because of a lack of time when compared to the other two segments. The Uninterested segment also differed significantly from the Weight conscious segment for social image and conventional eating motivations. The results indicated that the Uninterested segment is less motivated to choose foods because they are supposed to and because it would make them look good in front of others. The respondents in the Breakfast skippers segment reported levels of motivation for social image and conventional eating between the other two segments and there was no significant difference in these measures between the segments. The three segments differed in the levels of motivation for health, energy, opportunity, ability and weight control with Uninterested always indicating the lowest level of motivation for these measures, Breakfast skippers indicated a middle level of motivation and Weight conscious indicated the highest level of motivation for these measures. The mean differences are outlined in Table 5.

### 4.4. Associations between Eating Motivations and Healthful Behaviours

The drivers of eating behaviour may be indicated by the strongest motivations. However, healthful eating behaviours were not reported by all respondents. Examining the associations between motivations and healthful eating behaviour may indicate the motivators for healthful eating for each segment, and therefore offer insights on how each segment can be motivated towards healthier eating habits.

Relationships between the healthfulness of eating habits score were tested to examine which motivators were the strongest predictors of the healthfulness of eating behaviour score. Stepwise linear regression was performed to examine the relationships, optimizing the modelling procedure to acquire models with the most explanatory power with the least number of predictors, i.e., removing motivations that do not significantly contribute to the explanatory model. The model optimisation procedure was performed for each of the three segments.

The most parsimonious model for the Uninterested segment contained nine predictors, six of which were significant predictors (F(9, 178) = 7.62, *p* < 0.001, Adj R^2^ = 0.28). The model indicated that sociability (std β = −0.267, t = −3.563, *p* < 0.001), health (std β = −0.307, t = −4.175, *p* < 0.001), lack of time (std β = −0.203, t = −2.988, *p* = 0.003) and ability (std β = −0.177, t = −2.523, *p* = 0.013) were inversely related to healthfulness of eating habits score. This means that higher levels of these motivations predicted lower scores. Surprisingly, in this segment higher motivations to “eat what I eat…because it is healthy” and because “I can make many different things” (ability) were associated with less healthful eating habits. Furthermore, higher levels of motivation to eat foods because of lack of time and it is social predicted less healthful eating habits. Conversely, high motivation to eat to control weight (std β = 0.196, t = 2.756, *p* = 0.006) and “because it is trendy” (social norms) (std β = 0.183, t = 2.325, *p* = 0.021) predicted more healthful habits. Need for energy, convenience and hunger were included in the optimised model but contributed very little and were not significant predictors of healthful eating behaviour.

The most parsimonious model for the Breakfast skippers segment contained seven predictors, only one of which was significant (F (7, 85) = 3.61, *p* = 0.002, Adj R^2^ = 0.23). The significant predictor of healthful eating behaviour was social norms (std β = 0.288, t = 2.328, *p* = 0.022). Stronger motivations to “eat what I eat…because it is trendy” were associated with more healthful eating habits. Ability was also predictive of healthful eating but did not reach significance (std β = 0.220, t = 1.870, *p* = 0.065). Lack of time was predictive of less healthful eating habits (std β = −0.217, t = 1.746, *p* = 0.084) but also did not reach significance. Furthermore, weight control, convenience, social image and sociability were included in the optimised model but contributed very little and were not significant predictors of healthful eating behaviour.

The most parsimonious model for the Weight conscious segment contained eight predictors, three of which were significant (F (8, 202) = 4.92, *p* < 0.001, Adj R^2^ = 0.16). The model indicated higher motivations for eating due to lack of time was predictive of less healthful eating habits (std β = −0.230, t = 3.355, *p* = 0.001). Similarly, higher motivations for eating due to weight control predicted less healthful eating habits (std β = −0.144, t = −2.024, *p* = 0.044). Conversely, higher motivations for convenience predicted more healthful eating habits (std β = 0.142, t = 2.062, *p* = 0.041). Opportunity, ability, conventional eating, health and hunger were included in the optimised model but contributed very little to the model and were not significant predictors of healthful eating behaviour.

The relationships between the motivations and healthfulness of eating habits are summarised in Table 6 to indicate differences and similarities among the segments.

## 5. Discussion

Young adulthood is typified by major personal change that can result in the discarding and reforming of behavioural habits [51]. High levels of change are experienced by military trainees due to both emerging adulthood and training requirements [22]. This study found that there is room for improvement in the eating habits of both officer cadets and army recruits with army recruits reporting more healthful eating habits than officer cadets. Given the strong evidence of the importance of appropriate nutrition for both performance and health [2], this study supports recommendations for strategies to embed and strengthen healthful eating habits in early career military personnel.

Strategies developed for young adults to encourage healthful eating habits mainly focus on education, building skills and knowledge, or encouraging self-efficacy, self-regulation, or the desire to control weight [52,53,54]. Nevertheless, the evidence shows that educational interventions or information-based strategies are not very effective in changing eating habits [41,55]. Furthermore, strategies focusing on education, increasing knowledge and self-regulation are all individually focused and do not attempt to change the social and environmental influences surrounding the individual, which are known to have strong influences on eating behaviour [56]. Failure to address the surrounding environment influencing young adults going through a transformational life-stage can render individual focussed strategies ineffective [51].

The core principles of social marketing include the need to understand the individual and their experience to create effective strategies for behaviour change [57,58,59]. Therefore, this study examined the motivations for eating, revealing strong internal motivations such as the need for convenience, a desire for health, satiety, hunger and need for energy. Broader influences, both environmental and social, were examined but were not discovered to be as strong. There were differences between officer cadets and army recruits. Officer cadets had a significantly lower healthfulness of eating habits score (6.7) compared to army recruits (16.9). Given that the range of the healthfulness eating habits score is from 0 to 24, these means show that there is significant room for improvement in eating habits in both trainee groups. Furthermore, a large proportion of participants indicated never performing some of the beneficial behaviours, which is concerning. In general the army recruits expressed stronger motivations, specifically for satiety, convenience, because of ability, opportunity, for weight control and due to social norms. In two instances, however, the officer cadets expressed stronger motivations, namely, to eat for sociability and a lack of time.

Although the two trainee groups presented differences across eating motivations, segments were identified across the two sites. The trainee population revealed three distinct segments called Uninterested, Breakfast skippers and Weight conscious. According to the names, the members of these segments increase from left to right in their levels of motivation, opportunity and ability to eat healthfully and in healthful eating behaviour. Two of the segments (Uninterested and Weight conscious) were large and the third segment (Breakfast skippers) was about half the size of the other two segments. Even though the number of members in these segments differed, each segment had a substantial number of trainees from both pathways. Given that different segments were revealed, it is suggested that segment specific strategies can be designed that are better tailored to the needs, wants and preferences of each segment. In addition, this indicates that there might not be a need for pathway or location specific strategies, but instead a need for segment specific strategies that are transferrable across the different trainee populations.

Even though this study measured strong motivations for eating, the relevant findings for positively changing eating behaviours lie in the associations between motivations and healthfulness of eating habits. Consider the segment that reported the least healthful eating habits (Uninterested). Eating because food is perceived to be healthful was linked with less healthful habits, which might indicate either a misconception that the foods that are being eaten are healthful when in fact they are not, or little knowledge of what foods are actually healthful, or finally knowledge that a healthful diet should be consumed (and therefore reporting of high motivations for healthful eating) but failure to do so for a number of other reasons. Similarly, respondents in this segment reported eating foods because they have food preparation and cooking skills. However, they could be using these skills to prepare meals that are unhealthy. Eating for sociability was associated with less healthful habits as was a lack of time, which suggests that eating on social occasions, and a busy schedule might negatively affect healthful eating habits. However, expectations of others (or social norms) and eating to maintain a healthy weight contributed to healthier habits. These results show that accurate knowledge of healthful foods, skills to prepare healthful meals, and reinforcement of existing motivations to maintain a healthy weight and highlighting of the existing social norms is needed for the Uninterested segment. The Breakfast skippers segment might also benefit from supporting or increasing their ability to prepare meals as well as highlighting existing social norms, given both of these were associated with healthful eating behaviour. Interestingly, in the Weight conscious segment eating for weight control was associated with less healthful eating habits. This might be explained by a translation of weight concern into breakfast skipping or infrequent meal consumption. However, regular breakfast consumption might not be necessary for everyone given that the role of breakfast consumption in obesity prevention is still unknown [60]. Motivation to eat out of convenience was associated with healthful habits in the Weight conscious segment, indicating that respondents in this segment are able to prepare quick and healthful meals. Eating foods due to lack of time was associated with less healthful habits among all of the three segments, indicating a need for quick, healthful meals.

### Limitations and Future Research

This study is restricted by some limitations. First, a convenience cross-sectional sample was used representing young adult ADF trainees spread over two states of Australia. Therefore, generalisations beyond the current sample and the two training locations are challenging. Future studies should reach beyond this sample and collect data from early career ADF trainees across the country to establish key motivators for healthful eating and segments that are representative of early career ADF trainees.

The study used self-reported measures of behaviour. Self-reported measures are the most commonly used methods in social sciences [61] suffering from social desirability bias and possible incorrectness of data resulting from selective memory bias, and this needs to be taken into account [62]. Future research measuring actual eating behaviour through mechanical observations [63] could verify the accuracy of self-reported data, and observations should preferably be connected to individual self-reports to further strengthen the accuracy of the data.

This study sought to understand motivations and eating behaviours for military trainees using previously validated eating behaviour measures and scales [42] to measure self-reported behaviours that may contribute to healthful eating. Future studies may consider other measures that can provide finer detail on eating behaviours such as levels of fruit and vegetable intake; timing, frequency and size of meals; intake of foods in the context of a daily eating pattern. Additionally, future research extending understanding further may consider demographic factors such as age, sex and education, and geographic factors may extend understanding further.

The suggestions for strategies to positively change eating behaviour were based on the results without consulting the military trainees about their preferences and needs regarding solutions to encourage healthful eating. Future research utilising methods such as co-design are recommended to empower military trainees to accommodate their unique experiences into the program design process and contribute novel ideas [64,65]. Co-design methods have been successfully utilised across a variety of contexts and utilisation of co-design reduces the risk of new program failure, faster development times, and improved value and quality of developed programs [65,66,67].

Finally, the suggested strategies assume that creation of strategies tailored to each segment would produce better outcomes. Although evidence exists showing different segments respond to the same program differently [39], there is a lack of evidence that the application of segmentation improves program effectiveness. More research is recommended to determine whether a segmented program is more effective than a program where the entire target audience is treated the same way.

## 6. Conclusions

This study aimed to understand eating behaviours and factors influencing eating behaviour. The results indicated that strategies to increase healthful eating behaviours in all segments need to broaden beyond instilling or reinforcing health motivations and requiring self-regulation. One segment associated health negatively with eating habits, indicating that appealing to a desire for health is unlikely to be effective. However, there might be a need to offer information about healthful foods and what foods are actually healthful to change the perceptions about “apparently healthful” foods. Strategies also need to focus on the development of food and cooking skills within the trainee populations, and pair that with provision of social support and environments that encourage healthful eating.

## Figures and Tables

**Table 1:** Demographic profile of Australian Defence Force (ADF) trainees from two pathways.

	ADFA (Officer Cadets) (n = 195)	Kapooka (Army Recruits) (n = 297)	*p*
Gender *	**Frequency (%)**	**Frequency (%)**	**0.002**
Male	129 (66.2)	235 (79.1)	
Female	63 (32.3)	61 (20.5)	
Missing/not stated	3 (1.5)	1 (0.3)	
**Age** *			**0.000**
18–24	186 (95.4)	209 (70.4)	
25–29	7 (3.6)	56 18.9)	
30–34	1 (0.5)	17 (5.7)	
>35	1 (0.5)	15 (5.1)	
**Education**			**0.053**
High School	169 (86.7)	230 (77.4)	
Graduate Certificate	8 (4.1)	8 (2.7)	
Diploma	4 (2.1)	15 (5.1)	
Advanced Diploma	0 (0.0)	1 (0.3)	
Bachelor’s Degree	11 (5.6)	23 (7.7)	
Postgraduate Degree	0 (0.0)	6 (2.0)	
Other/Missing	3 (1.5)	14 (4.7)	
**BMI** *			**0.015**
<18.5 (less than normal)	2 (1.0)	3 (1.0)	
18.5–24.9 (normal)	142 (72.8)	178 (59.9)	
>24.9 (above normal)	51 (26.2)	98 (33.0)	
Missing	0 (0.0)	18 (6.1)	

* Significant at the 0.05 level or less. ADFA, Australian Defence Force Academy; BMI, Body Mass Index. The bold highlights the significant difference between the groups on a variable.

**Table 2:** Healthfulness of eating behaviours of ADF trainees.

		Officer Cadets (n = 195)	Army Recruits (n = 297)
Breakfast consumption ***		**Frequency (%)**	**Frequency (%)**
	Never	126 (65%)	8 (3%)
	Sometimes	41 (21%)	53 (18%)
	Often	25 (13%)	55 (19%)
	Always	3 (2%)	181 (61%)
Fruit consumption (2 portions/day) ***			
	Never	39 (20%)	21 (7%)
	Sometimes	46 (24%)	146 (49%)
	Often	96 (49%)	94 (32%)
	Always	14 (7%)	36 (12%)
Vegetable consumption (2 portions/day) ***			
	Never	92 (47%)	5 (2%)
	Sometimes	78 (40%)	67 (23%)
	Often	23 (12%)	130 (44%)
	Always	2 (1%)	95 (32%)
Cake/dessert consumption at meals ***			
	Never	42 (22%)	10 (3%)
	Sometimes	129 (66%)	42 (14%)
	Often	21 (11%)	185 (62%)
	Always	3 (2%)	60 (20%)
Wine/beer consumption at meals ***			
	Never	84 (43%)	5 (2%)
	Sometimes	102 (52%)	14 (5%)
	Often	8 (4%)	122 (41%)
	Always	1 (0%)	156 (53%)
Consumption of 3 meals/day ***			
	Never	115 (60%)	3 (1%)
	Sometimes	56 (29%)	46 (16%)
	Often	21 (11%)	79 (27%)
	Always	3 (2%)	169 (57%)
Dairy consumption ***			
	Never	42 (22%)	45 (15%)
	Sometimes	46 (24%)	115 (39%)
	Often	73 (37%)	73 (25%)
	Always	34 (17%)	64 (22%)
Water intake (at least 1–1.5L /day) ***			
	Never	118 (61%)	1 (0%)
	Sometimes	64 (33%)	27 (9%)
	Often	13 (7%)	88 (30%)
	Always	0 (0%)	181 (61%)

Significant differences between groups at *** *p* < 0.001 level.

**Table 3:** Constructs affecting motivations for eating.

		Officer Cadets (n = 195)	Army Recruits (n = 297)
Motivation (7-Point Scale)	Cronbach’s alpha Cadets, Recruits	Mean (SD)	Mean (SD)
Health/balanced diet (3 survey items)	0.83, 0.88	4.96 (1.32)	5.10 (1.39)
Convenience (3 survey items) **	0.68, 0.73	4.63 (1.25)	4.93 (1.09)
Ability (4 survey items) ***	0.74, 0.84	3.40 (1.32)	4.35 (1.52)
Opportunity (3 survey items) ***	0.68, 0.79	3.38 (1.27)	4.24 (1.51)
Sociability (3 survey items) **	0.80, 0.82	3.73 (1.31)	3.37 (1.34)
Weight control (3 survey items) *	0.75,0.78	3.16 (1.33)	3.45 (1.36)
Social norms (2 survey items) **	0.65, 0.60	2.52 (1.21)	2.83 (1.36)
Social image (3 survey items)	0.78, 0.62	2.11 (1.03)	2.16 (1.02)
Need for energy (1 survey item)	n/a	5.13 (1.8)	5.40 (1.64)
Hunger (1 survey item)	n/a	5.18 (1.54)	5.15 (1.54)
Satiety (1 survey item) ***	n/a	4.57 (1.63)	5.20 (1.25)
Conventional eating (1 survey item)	n/a	4.16 (1.68)	4.38 (1.70)
Lack of time (1 survey item) ***	n/a	4.25 (1.90)	2.82 (1.54)

Significant differences between groups at * *p* < 0.05, ** *p* < 0.01, or *** *p* < 0.001 level, n/a = not applicable.

**Table 4:** Three segment solution—demographic and behavioural variables.

	Uninterested (n = 188)	Breakfast skippers (n = 93)	Weight conscious (n = 211)	*p*
**Gender**				0.153
Male	76.6%	65.6%	75.4%	
Female	23.4%	33.3%	23.2%	
Missing/not stated	0%	1.1%	1.4%	
Age *				**0.000**
18–24	92.6%	50.5%	82.5%	
25–29	5.9%	25.8%	13.3%	
30–34	1.1%	14%	1.4%	
>35	0.5%	9.7%	2.8%	
Education *				**0.000**
High School	100%	0%	100%	
Graduate Certificate	0%	17.2%	0%	
Diploma	0%	20.4%	0%	
Advanced Diploma	0%	1.1%	0%	
Bachelor’s Degree	0%	36.6%	0%	
Postgraduate Degree	0%	6.5%	0%	
**BMI**				0.633
<18.5 (less than normal)	1.1%	0%	1.4%	
18.5–24.9 (normal)	68.6%	61.3%	63.5%	
>24.9 (above normal)	26.1%	35.5%	31.8%	
Missing	4.3%	3.2%	3.3%	
Healthfulness of eating habits score *	10.4 (4.8)	13.9 (5.4)	14.5 (6.2)	**0.000**
You eat breakfast *	1.2 (1.1)	1.9 (1.2)	1.9 (1.2)	**0.000**
You eat at least 2 portions (200 g) of fruit	1.4 (0.8)	1.5 (0.8)	1.5 (0.9)	0.744
You eat at least 2 portions (200 g) of vegetables *	1.2 (0.9)	1.7 (0.9)	1.7 (1.0)	**0.000**
You eat cake or a dessert at meals *	1.3 (0.8)	1.6 (0.9)	1.8 (0.8)	**0.000**
You drink wine or beer at meals *	1.3 (1.1)	2.0 (1.0)	2.0 (1.1)	**0.000**
You eat 3 meals *	1.2 (1.1)	1.9 (1.1)	1.9 (1.2)	**0.000**
You drink at least one glass of milk or you eat at least one cup of yoghurt	1.5 (1.0)	1.5 (1.0)	1.6 (1.0)	0.677
You drink at least 1–1.5 L of water *	1.2 (1.1)	1.9 (1.1)	2.1 (1.2)	**0.000**

* Significant at the 0.05 level or less. The bold highlights the significant difference between the groups on a variable.

**Table 5:** Differences in eating motivations between the segments.

Motivations for Eating	Uninterested (n = 188)	Breakfast Skippers (n = 93)	Weight Conscious (n = 211)
	Mean	Mean	Mean
Need for energy	4.7 ^a^	5.3 ^b^	5.8 ^c^
Hunger	5.1 ^a^	5.1 ^a^	5.3 ^a^
Health/balanced diet	4.1 ^a^	5.1 ^b^	5.8 ^c^
Satiety	4.5 ^a^	5.4 ^b^	5.0 ^b^
Convenience	4.7 ^a^	4.9 ^a^	4.7 ^a^
Conventional eating	3.9 ^a^	4.3 ^a b^	4.6 ^b^
Ability	2.9 ^a^	4.3 ^b^	4.8 ^c^
Opportunity	2.8 ^a^	4.1 ^b^	4.8 ^c^
Sociability	3.4 ^a^	3.4 ^a^	3.7 ^a^
Lack of time	4.1 ^a^	3.0 ^b^	2.9 ^b^
Weight control	2.7 ^a^	3.4 ^b^	3.9 ^c^
Social norms	2.5 ^a^	2.8 ^a^	2.8 ^a^
Social image	2.0 ^a^	2.1 ^a b^	2.3 ^b^

Segment differences (*p* < 0.05) are marked with superscript letters ^a b^ or ^c^ Means in the same row that are marked with the same letter are not significantly different.

**Table 6:** Summary of associations between healthfulness of eating habits score and motivations across the segments.

	Uninterested (n = 188)	Breakfast Skippers (n = 93)	Weight Conscious (n = 211)
Need for energy			
Hunger			
Health/balanced diet	Negative (−4.175)		
Satiety			
Convenience			Positive (2.062)
Conventional eating			
Ability	Negative (−2.523)		
Opportunity			
Sociability	Negative (−3.563)		
Lack of time	Negative (−2.988)		Negative (−3.355)
Weight control	Positive (2.756)		Negative (−2.024)
Social norms	Positive (2.325)	Positive (2.328)	
Social image			

## Appendix A

### Motivation Questions

Motivation questions included nine constructs with three to four items per construct measured on a seven-point Likert scale (1 = Never, 2 = Rarely, 3 = Occasionally, 4 = Sometimes, 5 = Frequently, 6 = Usually and 7 = Always).

Need and Hunger

- I eat what I eat…because I’m hungry
- I eat what I eat…because I need energy
- I eat what I eat…because it is pleasantly filling

Health

- I eat what I eat…to maintain a balanced diet
- I eat what I eat…because it is healthy
- I eat what I eat…because it keeps me in shape (e.g., energetic, motivated)

Weight Control

- I eat what I eat…because it is low in calories
- I eat what I eat…because I watch my weight
- I eat what I eat…because it is low in fat

Convenience

- I eat what I eat…because it is easy to prepare
- I eat what I eat…because it is quick to prepare
- I eat what I eat…because it is the most convenient

Sociability

- I eat what I eat…because it makes social gatherings more comfortable
- I eat what I eat…so that I can spend time with other people
- I eat what I eat…because it is social

Social Norms

- I eat what I eat…to avoid disappointing someone who is trying to make me happy
- I eat what I eat…because it is trendy
- I eat what I eat…because I am supposed to eat it

Social Image

- I eat what I eat…because it makes me look good in front of others
- I eat what I eat…because it would be impolite not to eat it
- I eat what I eat…because others like it

Ability

- I eat what I eat…because I have the skills to shop for my own food
- I eat what I eat…because I can make many different things
- I eat what I eat…because I can cook many different things
- I eat what I eat…because I have a good level of knowledge of the various ways to cook food

Opportunity

- I eat what I eat…because there are lots of different fruit and vegetables available
- I eat what I eat…because there are many shops selling fruit and vegetables nearby
- I eat what I eat…because fruit and vegetables are easy to buy
- I eat what I eat…because I don’t have the time to shop for my own food
